# Immunogenicity and safety of 3-dose primary vaccination with combined DTPa-HBV-IPV/Hib in Indian infants

**DOI:** 10.1080/21645515.2016.1225639

**Published:** 2016-09-15

**Authors:** Sanjay K. Lalwani, Sharad Agarkhedkar, Balasubramanian Sundaram, Niranjana S. Mahantashetti, Nandini Malshe, Shalaka Agarkhedkar, Olivier Van Der Meeren, Shailesh Mehta, Naveen Karkada, Htay Htay Han, Narcisa Mesaros

**Affiliations:** a Bharati Vidyapeeth Deemed University Medical College, Pune, India; b Dr D Y Patil Medical College, Pune, India; c Kanchi Kamakoti Childs Trust Hospital and CHILDS Trust Medical Research Foundation, Chennai, India; d KLE University's J.N. Medical College, Belgaum, India; e GSK Vaccines, Wavre, Belgium; f GSK Pharmaceuticals, Bangalore, India; g GSK Vaccines, King of Prussia, USA

**Keywords:** combination vaccines, recombinant DTPa-HBV-IPV/Hib, seropositive, seroprotection, vaccine associated paralytic polio

## Abstract

Multivalent combination vaccines have reduced the number of injections and therefore improved vaccine acceptance, timeliness of administration and global coverage. The hexavalent diphtheria-tetanus-acellular pertussis-hepatitis B-inactivated poliovirus/*Haemophilus influenzae* type b (DTPa-HBV-IPV/Hib; *Infanrix hexa*™) vaccine, administered according to various schedules, is widely used for the primary vaccination of infants worldwide. In the current publication, we are presenting the immunogenicity and safety of 3 doses of DTPa-HBV-IPV/Hib vaccine when administered to Indian infants. 224 healthy infants (mean age 6.8 weeks) were vaccinated at 6–10–14 weeks (W) of age (n = 112) or 2–4–6 months (M) of age (n = 112). One month after the third vaccine dose, the seroprotection/seropositivity status against diphtheria, pertussis, tetanus, polio, hepatitis B and Hib antigens ranged from 98.6% to 100% in both groups. The vaccine response rate to the pertussis antigens ranged from 97% to 100%. Pain (6–10–14W group: 25.2%; 2–4–6M group: 13.4%) and fever (15.3% and; 15.2%, respectively) were the most frequently reported solicited local and general symptoms. Unsolicited adverse events were reported for 35.7% (6–10–14W group) and 22.3% (2–4–6M group) of subjects. No vaccine related serious adverse events were reported. In conclusion, the hexavalent DTPa-HBV-IPV/Hib vaccine was immunogenic and well tolerated, irrespective of the dosing schedule.

## Introduction

Infections resulting from vaccine-preventable diseases including diphtheria, tetanus, pertussis, *Haemophilus influenzae* type B (Hib) and poliovirus, account for a large number of deaths in children below 5 y of age.^1,2^ Infants and children infected with hepatitis B are at risk to become chronic carriers of HBV, with potential complications such as cirrhosis and hepatocellular carcinomas later in life.^3^ It is estimated that 18.7 million infants worldwide and >4 million infants in India did not receive vaccination against diphtheria-tetanus-pertussis (DTP) during their first year of life.^4^

Combination vaccines, such as DTP, have been widely adopted in pediatric vaccination programs, as the reduced number of injections and simplified administration has been associated with higher compliance and improved vaccine coverage.^5,6^ A combined hexavalent diphtheria-tetanus-acellular pertussis-hepatitis B-inactivated poliomyelitis and Hib conjugate vaccine (DTPa-HBV-IPV/Hib, *Infanrix hexa*™, GSK Vaccines, Belgium) has been widely used for over 15 y.^7,8^ Many studies have established the immunogenicity and safety of the combined DTPa-HBV-IPV/Hib administered according to different primary vaccination schedules and in diverse settings.^5,7,9-11^ Furthermore, comparable efficacy and safety profiles to those of the respective monovalent components have also been established.^7,10^

In accordance with the Expanded Program on Immunization (EPI) recommendations, the Indian infant vaccination schedule comprises primary vaccination with 3 doses of DTP at 6, 10 and 14 weeks, followed by a booster dose in the second year of life.^12^ In addition, the Indian Academy of Pediatrics (IAP) recommends that infants are vaccinated against hepatitis B at birth, and 6 and 14 weeks of age; against Hib infections at 6, 10, and 14 weeks of age; oral poliovirus vaccine (OPV) should be given at birth and inactivated poliovirus vaccine (IPV) at 6, 10 and 14 weeks of age.^13^ In 2015, the coverage with 3 doses of DTP, Hib, hepatitis B and polio vaccines was estimated at 87%, 45%, 87%, and 86%, respectively, in India.^14^

Currently, only pentavalent combination vaccines are available in India (i.e. DTPw-whole cell pertussis [Pw]-HBV-Hib and DTPa-IPV/Hib).

Although the use of OPV in India has led to the country being declared polio-free in 2014,^15^ it also contributes to the risk of vaccine-associated paralytic poliomyelitis (VAPP).^16,17^ The incidence of VAPP has been estimated at 2–4 cases/million birth cohort per year in countries using OPV^18^ which highlights the need to consider the risks associated with continued OPV use.^19^ Studies have reported the incidence of VAPP cases associated with OPV in India^16^ and have recommended replacing OPV with IPV in order to avoid VAPP.^20,21^ Moving from OPV to IPV is very important in complete global eradication of polio, and vaccines combining IPV (e.g. DTPa-HBV-IPV/Hib) appear to be the most convenient way to facilitate this change.^22^

In order to support policy makers and healthcare providers to make informed decisions on vaccination in India, we evaluated the immunogenicity and safety of DTPa-HBV-IPV/Hib when administered to Indian infants according to either the 6–10–14 weeks (W) of age or 2–4–6 months (M) of age schedule.

## Results

### Study population

**Figure 1.**
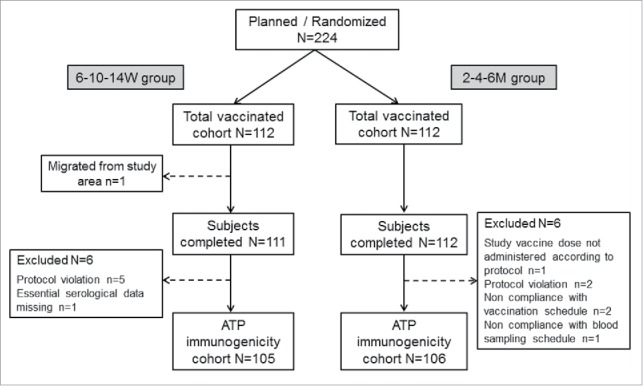
Study flow chart.

A total of 224 children were enrolled and vaccinated (total vaccinated cohort [TVC]) in this study, of whom 223 (6–10–14W group n = 111; 2–4–6M group n = 112) completed the study (Fig. 1). 211 subjects (6–10–14W group n = 105; 2–4–6M group n = 106) were included in according-to-protocol (ATP) immunogenicity cohort. Both groups were comparable demographically: all subjects were of Central/South Asian heritage with an overall mean age of 6.8 (±1.1) weeks; 53.6% were male.

### Assessment of immunogenicity

One month after the third vaccine dose, seropositivity/seroprotection rates were similar in both groups (Table 1). All subjects in both groups were seroprotected against diphtheria and tetanus.

**Table 1.** Seroprotection/seropositivity rates and GMC/Ts for anti-diphtheria, anti-tetanus, anti-PRP, anti-HBs, anti-poliovirus 1, 2, 3, anti-PT, anti-FHA and anti-PRN antibodies by group before first dose and one month after the third vaccine dose (ATP cohort for immunogenicity).

			6–10–14W group						2–4–6M group					
			Seroprotection/seropositivity				GMC/T	95% CI	Seroprotection/seropositivity				GMC/T	95% CI
Antibody		Time-point	N	n	%	95% CI			N	n	%	95% CI		
Anti-diphtheria		Pre	—	—	—	—	—	—	—	—	—	—	—	—
(≥ 0.1 IU/ml)		Post	105	105	100	96.5–100	2.334	2.049–2.659	106	106	100	96.6–100	3.726	3.260–4.258
Anti-tetanus (≥ 0.1 IU/ml)		Pre	—	—	—	—	—	—	—	—	—	—	—	—
		Post	105	105	100	96.5–100	3.307	2.925–3.739	106	106	100	96.6–100	4.904	4.378–5.493
Anti-PRP (≥ 0.15 μg/ml)		Pre	—	—	—	—	—	—	—	—	—	—	—	—
		Post	105	104	99.0	94.8–100	2.697	2.176–3.343	106	105	99.1	94.9–100	5.404	4.168–7.006
Anti-HBs (≥ 10 mIU/ml)		Pre	80	14	17.5	9.9–27.6	5.9	4.2–8.3	81	13	16.0	8.8–25.9	5.6	4.1–7.7
		Post	101	101	100	96.4–100	1695.7	1395.2–2060.9	105	104	99.0	94.8–100	3314.5	2645.2–4153.1
Anti-poliovirus (≥ 1:8)	Type 1	Pre	97	71	73.2	63.2–81.7	53.5	33.5–85.3	102	70	68.6	58.7–77.5	31.9	21.5–47.2
		Post	99	99	100	96.3–100	884.3	666.6–1173.1	99	99	100	96.3–100	1799.2	1429.0–2265.5
	Type 2	Pre	56	38	67.9	54.0–79.7	32.5	19.2–55.0	55	42	76.4	63.0–86.8	36.2	22.2–59.0
		Post	77	77	100	95.3–100	840.2	616.9–1144.2	88	88	100	95.9–100	2138.7	1658.1–2758.7
	Type 3	Pre	88	23	26.1	17.3–36.6	8.0	5.9–10.8	91	30	33.0	23.5–43.6	11.9	8.3–17.0
		Post	74	73	98.6	92.7–100	923.7	691.9–1233.2	79	79	100	95.4–100	2245.5	1866.1–2702.1
Anti-pertussis (≥5 EL.U/ml)	PT	Pre	105	44	41.9	32.3–51.9	5.0	4.2–6.0	104	37	35.6	26.4–45.6	4.6	3.8–5.6
		Post	105	105	100	96.5–100	107.3	96.6–119.1	106	106	100	96.6–100	108.2	97.4–120.2
	FHA	Pre	101	89	88.1	80.2–93.7	18.7	15.0–23.3	102	90	88.2	80.4–93.8	20.1	16.1–25.2
		Post	105	105	100	96.5–100	293.7	259.4–332.6	106	106	100	96.6–100	369.3	335.5–406.5
	PRN	Pre	105	19	18.1	11.3–26.8	3.4	2.9–3.9	104	15	14.4	8.3–22.7	3.2	2.8–3.7
		Post	105	105	100	96.5–100	224.4	194.2–259.3	106	106	100	96.6–100	243.6	213.2–278.4

N: number of subjects with available results; n/%: number/percentage of subjects with concentration ≥ required threshold; GMC/T: geometric mean antibody concentrations / titers; 95% CI: 95% confidence interval; Pre: blood sample collected before the first dose of the primary vaccination course; Post: blood sample collected one month after the third dose of the primary vaccination course; PRP: Hib capsular polysaccharide polyribosyl-ribitol phosphate; HBs: Hepatitis B surface antigen; PT: pertussis toxoid; FHA: filamentous haemagglutinin; PRN: pertactin.

In both groups, ≥99.0% of subjects were seroprotected against Hib polyribosylribitol phosphate (PRP) (Table 1).

Before the first dose study vaccination, 17.5% of the subjects in 6–10–14W group and 16.0% of the subjects in 2–4–6M group were seroprotected for hepatitis B, reflecting the hepatitis B birth vaccination that all study participants received. One month after primary vaccination, ≥99.0% of subjects were seroprotected against hepatitis B in both groups.

Before the first dose of study vaccination, at least 68.6%, 67.9% and 26.1% of the subjects were seroprotected against poliovirus type 1, 2 and 3, respectively, in both groups, reflecting the OPV vaccination all study participants received at birth. One month post-primary vaccination, all subjects were seroprotected against poliovirus 1 and 2 in both groups; 98.6% subjects in 6–10–14W group (all except one subject) and all subjects in 2–4–6M group were seroprotected against poliovirus 3 (Table 1).

One month post-primary vaccination, all subjects in both groups were seropositive for anti-pertussis toxin (PT), anti-filamentous haemagglutinin (FHA) and anti-pertactin (PRN) antibodies (≥5 EL.U/ml) and the vaccine response rates to the pertussis antigens were above 97% and 98% in the 6–10–14W and 2–4–6M groups, respectively, for at least one antigen (Table 2). At pre-vaccination, the geometric mean concentration (GMC) values were 5.0 EL.U/ml and 4.6 EL.U/ml for anti-PT; 18.7 EL.U/ml and 20.1 EL.U/ml for anti-FHA; and 3.4 EL.U/ml and 3.2 EL.U/ml for anti-PRN in the 6–10–14W and 2–4–6M groups, respectively. The levels of pertussis antibodies observed at the pre-vaccination are probably due to the natural exposure to the disease or maternal transfer of antibodies against pertussis. One month after the third dose, the GMC values were 107.3 EL.U/ml and 108.2 EL.U/ml for anti-PT; 293.7 EL.U/ml and 369.3 EL.U/ml for anti-FHA; and 224.4 EL.U/ml and 243.6 EL.U/ml for anti-PRN across the 2 groups.

**Table 2.** Vaccine response rate (%) to anti-PT, anti-FHA and anti-PRN antibody concentrations one month after third vaccine dose (ATP cohort for immunogenicity).

	% Vaccine response (95% CI)	
Antibody	6–10–14W group	2–4–6M group
Anti-PT antibody	100 (96.5–100)	99.0 (94.8–100)
Anti-FHA antibody	97.0 (91.6–99.4)	98.0 (93.1–99.8)
Anti-PRN antibody	99.0 (94.8–100)	99.0 (94.8–100)

95% CI: 95% confidence interval; PT: pertussis toxoid; FHA: filamentous haemagglutinin; PRN: pertactin.

### Assessment of safety and reactogenicity

**Figure 2.**
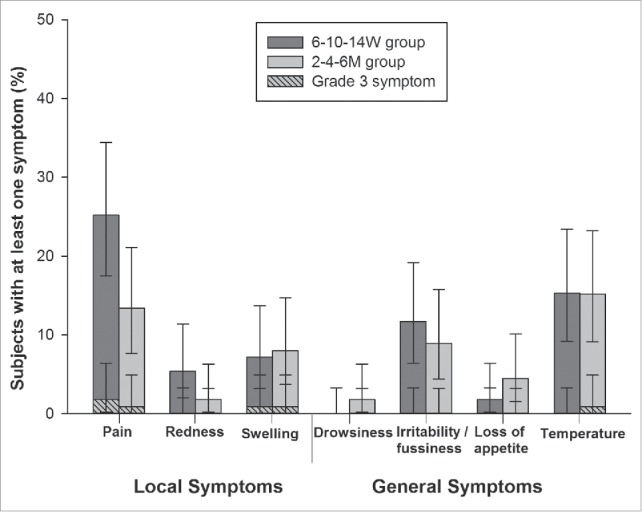
Overall incidence of solicited local and general symptoms for 4 d after primary vaccination (Total vaccinated cohort).

During the 4-day follow-up period, pain was the most frequently reported solicited local symptom, being reported in 25.2% (28/111) and 13.4% (15/112) subjects in the 6–10–14W and 2–4–6M groups, respectively (Fig. 2). Pain was also the most frequently reported grade 3 local symptom (1.8% of subjects in the 6–10–14W group; 0.9% of subjects in the 2–4–6M group). Fever (axillary temperature ≥37.5°C) was the most frequently reported solicited general symptom in both groups, with an incidence of 15.3% (17/111 subjects) and 15.2% (17/112 subjects) in the 6–10–14W and 2–4–6M groups, respectively. In all instances, fever was assessed as causally related to the vaccination. It was also the most frequently reported grade 3 (i.e., axillary temperature >39°C) solicited general symptom and was recorded in 0.9% of subjects in the 2–4–6M group. None of the subjects in the 6–10–14W group reported grade 3 fever.

During the 31-day follow-up period, at least one unsolicited adverse event (AE) was reported for 35.7% (40/112) and 22.3% (25/112) of subjects in 6–10–14W and 2–4–6M groups, respectively. Upper respiratory tract infection was the most frequently reported unsolicited AE (6–10–14W group: 16.1% [18/112]; 2–4–6M group: 9.8% [11/112]). None of the subjects reported grade 3 unsolicited AEs. Three serious AEs (SAEs; lower respiratory tract infection, pneumonia and bronchiolitis) were reported in 5 subjects in both groups (6–10–14W group, n = 2; 2–4–6M group, n = 3). None of the unsolicited adverse events or SAEs were assessed by the investigator as causally related to the vaccination. No subjects withdrew from the study due to an AE or SAE.

## Discussion

Since its licensure in 2000, more than 137 million doses of the combined DTPa-HBV-IPV/Hib vaccine were distributed worldwide. Post-marketing surveillance data confirmed its clinically acceptable safety profile and its robust immuno-genicity.^5,8,23^

We assessed the immunogenicity and safety of primary vaccination with the combined DTPa-HBV-IPV/Hib vaccine administered to Indian infants according to either a 6–10–14W or 2–4–6M schedule. One month after third vaccine dose, the seroprotection rates ranged from 98.6% to 100% for all study antigens. These results are in line with a previous study from the Philippines where seroprotection rates ranging from 90% to 100% were recorded after primary vaccination with the DTPa-HBV-IPV/Hib vaccine using the 6–10–14W schedule.^11^ Likewise, several studies have reported comparable seroprotection/seropositivity rates one month post primary vaccination according to a 2–4–6M schedule.^24–26^ The vaccine response to the pertussis antigens (97–100%) was also similar to previous reports.^24^

Post-primary vaccination, ≥99.0% of the subjects were seroprotected against anti-HBs. Even though it has been established that the hepatitis B immunogenicity elicited by the combined DTPa-HBV-IPV/Hib vaccine is comparable to that elicited by the monovalent vaccine,^27^ previous studies have demonstrated that a HBV birth dose is essential to achieve targeted seroprotection rates ≥95%, with the combined vaccine, when the accelerated 6–10–14W schedule is used.^11^ It should be noted that, in accordance with local Indian recommendations, all subjects included in this study received a birth dose of hepatitis B vaccine.

Similarly, the pre-vaccination seroprotection rates for poliovirus 1, 2 and 3 were due to the birth dose of OPV received as per local standard of care. Post-vaccination seroprotection rates (98.6% to 100%) were in line with previous reports.^11^ Cases of VAPP are occasionally reported in countries where wild poliovirus has been eliminated, and therefore replacing OPV by IPV in pediatric immunization schedules is an important step towards global polio eradication.^22^ The role of IPV in poliovirus control and complete polio eradication is reflected in WHO guidelines recommending the administration of at least one IPV dose to all children, and by the growing number of countries transitioning from OPV to an all-IPV schedule.^18^ The combined DTPa-HBV-IPV/Hib vaccine would facilitate this transition from OPV to IPV in India, which is a key step in the fight for global polio elimination.

The combined DTPa-HBV-IPV/Hib vaccine was generally well tolerated by Indian infants. This vaccine was known to be associated with low incidence of clinically significant AEs and very rare SAEs.^5^ There were no withdrawals from the study due to any AEs or SAEs. A previous study (NCT00316147) has also confirmed the safety of the hexavalent vaccine in Indian infants when administered according to either recommended 6–10–14W or internationally adopted 2–4–6M vaccination schedules.^28^ These findings are in line with previous reports where acceptable safety profiles have been observed across different dosing schedules.^11,23^

Our study is the first to report the immunogenicity and safety of DTPa-HBV-IPV/Hib in India. A high level of compliance was observed, with 99.6% subjects receiving all the 3 vaccine doses and 94.2% subjects being eligible for inclusion in the immunogenicity cohort. Although our study was limited by the small sample size and exploratory nature of analysis, it did comply with the current regulatory requirements in India.

Combined vaccines are the preferred choice for infant immunization as they simplify the childhood vaccination schedule. Furthermore, they are convenient and cost-effective, with better coverage, improved compliance, and require fewer clinic visits.^6,29^ The choice of dosing schedule differs markedly between countries, although the extended 2–4–6M schedule has the advantage of facilitating visits at the crucial 4 and 6 month stages, when infants are being weaned (from breastfeeding).^30^ This schedule is widely adopted in Western countries, as well as in some Asian countries.^31^

Recent resurgence in pertussis disease incidence and a shift of the disease to older age groups in some countries that switched from DTPw to DTPa vaccination^32^ in infancy, were partly attributed to waning of long-term Pa vaccine efficacy, which was estimated to be more rapid when compared to Pw vaccines.^33^ Therefore, WHO endorsed the use of DTPw in settings in which additional booster immunizations or other strategies for preventing early pertussis infant mortality (e.g., pertussis maternal immunization) are not easily attained.^34^ Although the reactogenicity of DTPa vaccines was shown to be more reduced than that of DTPw, the currently available measures of immune response against pertussis might not be predictive of long-term effectiveness, and so interchangeability of infant DTP vaccines should be further explored and cautiously approached in the context of different pediatric immunization programs.

In conclusion, the combined DTPa-HBV-IPV/Hib vaccine is immunogenic and well tolerated when administered according to either a 6–10–14W or 2–4–6M schedule to healthy Indian infants. DTPa-HBV-IPV/Hib might be considered a viable choice in the transition from OPV to IPV vaccination, which could contribute to reducing the risk of VAPP.

## Materials and methods

### Study design and participants

This open-label, phase III study was conducted at 4 Indian centers between 16 April 2012 and 25 February 2013 (www.clinicaltrials.gov NCT01353703). It was conducted according to Good Clinical Practice and the Declaration of Helsinki, and the study protocol was approved by the ethics committees at all participating study centers. Written informed consent was obtained from the parents/guardians of all the subjects before initiating any study-related procedure.

A target enrollment of 224 subjects (112 subjects per group) was considered, which after a 10% drop-out would still have ensured 100 subjects per group evaluable for immunogenicity. Subjects were randomized (1:1) in 2 groups to receive 3 doses of the combined DTPa-HBV-IPV/Hib vaccine according to either a 6–10–14W or 2–4–6M schedule (control group). Healthy infants aged 6 to 10 weeks, who had previously received a dose of hepatitis B vaccine within the first week of birth, and born after a gestation period of 36 weeks, were enrolled in this study. The exclusion criteria included: use of any investigational drug/vaccine (except human rotavirus) 30 d before vaccination or any immunoglobulins/blood products since birth, or immunosuppressants/immune modifying drugs within 6 months of the first vaccine dose, known hypersensitivity to the study vaccine components, confirmed/suspected immunosuppressive/immunodeficient condition, family history of congenital/hereditary immunodeficiency, major congenital defects or chronic illness and/or fever at the time of enrolment. Other reasons for exclusion included previous exposure to diphtheria, tetanus, pertussis, hepatitis B, poliomyelitis and/or Hib vaccination or disease (with the exception of a birth dose of hepatitis B and OPV vaccination).

### Study vaccine

Each 0.5 ml dose of the recombinant DTPa-HBV-IPV/Hib vaccine contained ≥30 international units (IU) of diphtheria toxoid (DT), ≥40 IU tetanus toxoid (TT), 25µg PT, 25µg FHA, 8µg PRN, 10µg recombinant hepatitis B surface antigen, 40D, 8D and 32D antigen units of poliovirus types 1, 2 and 3, respectively, and 10µg PRP conjugated to TT (20–40µg). All vaccine doses were administered intramuscularly into the upper side of the right thigh.

### Immunogenicity assessment

Blood samples were collected from all subjects before the first dose (pre-vaccination) and one month after the third dose (post-vaccination). Antibodies against DT, TT, PRP, FHA, and PRN were measured using standard in-house enzyme-linked immunosorbent assays (ELISA). The anti-HBs concentrations were measured using a commercial chemiluminescence assay (*ADIVA Centaur*™ Anti-HBs, Siemens Healthcare, Marburg, Germany; cut-off: 6.2 mIU/mL). Antibodies against each poliovirus (type 1, 2 and 3) were measured by a virus micro-neutralization assay. Seroprotection was defined as an antibody concentration ≥0.1 IU/ml for diphtheria and tetanus, ≥10 mIU/ml for antibodies to hepatitis B (anti-HBs), ≥1:8 dilution for poliomyelitis, and ≥0.15µg/ml for PRP. Seropositivity for antibodies against pertussis antigens (PT, FHA and PRN) was defined as an antibody concentration ≥5 EL.U/ml. The primary objective of the study was to assess the vaccine response rates to the pertussis antigens and seroprotection rates against all other antigens included in the vaccine, one month after the third dose.

### Assessment of safety and reactogenicity

All and grade 3 solicited local (injection site pain, redness, swelling) and general (drowsiness, fever, irritability/fussiness and loss of appetite) symptoms were recorded for 4 (Day 0–3) subsequent days after each vaccine dose. Unsolicited AEs were recorded for 31 (Day 0–30) days after vaccination. SAEs were recorded during the entire study period.

### Statistical analysis

The statistical analyses were performed using SAS (Statistical Analysis Software, SAS Institute Inc., Cary, NC, United States).

The primary analyses for immunogenicity were performed on the ATP cohort. It included subjects meeting all eligibility criteria, complying with the procedures and intervals defined in the protocol.

The GMC/geometric meant titer calculations were performed by taking the anti-log of the mean of the log transformations. Seropositivity rates against PT, FHA and PRN; seroprotection rates against HBs, DT, TT, PRP antigen and poliovirus types 1, 2 and 3 with 95% confidence intervals (CI) were calculated. Percentage of subjects with anti-diphtheria and anti-tetanus antibody concentrations ≥1.0 IU/ml; anti-HBs antibody concentrations ≥100 mIU/ml; anti-PRP antibody concentrations ≥1.0 μg/ml were calculated with 95% CI. GMC/Ts with 95% CI were tabulated for antibodies against each antigen. Vaccine response rates with exact 95% CI to PT, FHA and PRN were calculated one month after the third vaccine dose.

The analyses for safety and reactogenicity were performed on the TVCs, which included all subjects who had received at least one dose of the study vaccine. The percentage of subjects with at least solicited/unsolicited AE were tabulated with exact 95% CI after each vaccine dose and overall.

## Trademark statement

*Infanrix hexa* is trademark of the GSK group of companies.

Centaur is a trademark of Siemens Healthcare.

## Abbreviations

Anti-HBs: antibodies to hepatitis B surface antigen
ATP: according-to-protocol
CI: confidence interval
DT: diphtheria toxoid
DTPa: diphtheria-tetanus-acellular pertussis vaccine
DTPa-HBV-IPV/Hib: combined diphtheria-tetanus-acellular pertussis-hepatitis B-inactivated poliovirus and *Haemophilus influenzae* type b vaccine
DTPw: diphtheria-tetanus-whole cell pertussis vaccine
EL.U: ELISA units
ELISA: enzyme-linked immunosorbent assay
FHA: filamentous haemagglutinin
GMC: geometric mean concentration
GMT: geometric mean titer
Hib: *Haemophilus influenzae* type b
IPV: inactivated poliovirus vaccine
IU: international unit(s)
mIU: milli-international units
OPV: oral poliovirus vaccine
PRN: pertactin
PRP: Hib capsular polysaccharide polyribosyl-ribitol phosphate
PT: pertussis toxoid
TT: tetanus toxoid
TVC: total vaccinated cohort
ED_50_: median effective dose
VAPP: vaccine associated paralytic polio
